# Van der Waals Integrated Silicon/Graphene/AlGaN Based Vertical Heterostructured Hot Electron Light Emitting Diodes

**DOI:** 10.3390/nano10122568

**Published:** 2020-12-21

**Authors:** Nallappagari Krishnamurthy Manjunath, Chang Liu, Yanghua Lu, Xutao Yu, Shisheng Lin

**Affiliations:** 1 College of Microelectronics, Zhejiang University, Hangzhou 310027, China; manjunath@zju.edu.cn (N.K.M.); 12031105@zju.edu.cn (C.L.); luyanghua6@zju.edu.cn (Y.L.); 21931041@zju.edu.cn (X.Y.); 2 College of Information Science and Electronic Engineering, Zhejiang University, Hangzhou 310027, China; 3 State Key Laboratory of Modern Optical Instrumentation, Zhejiang University, Hangzhou 310027, China

**Keywords:** van der Waals contact, graphene, hetero-structure, light emitting diode

## Abstract

Silicon-based light emitting diodes (LED) are indispensable elements for the rapidly growing field of silicon compatible photonic integration platforms. In the present study, graphene has been utilized as an interfacial layer to realize a unique illumination mechanism for the silicon-based LEDs. We designed a Si/thick dielectric layer/graphene/AlGaN heterostructured LED via the van der Waals integration method. In forward bias, the Si/thick dielectric (HfO_2_-50 nm or SiO_2_-90 nm) heterostructure accumulates numerous hot electrons at the interface. At sufficient operational voltages, the hot electrons from the interface of the Si/dielectric can cross the thick dielectric barrier via the electron-impact ionization mechanism, which results in the emission of more electrons that can be injected into graphene. The injected hot electrons in graphene can ignite the multiplication exciton effect, and the created electrons can transfer into p-type AlGaN and recombine with holes resulting a broadband yellow-color electroluminescence (EL) with a center peak at 580 nm. In comparison, the n-Si/thick dielectric/p-AlGaN LED without graphene result in a negligible blue color EL at 430 nm in forward bias. This work demonstrates the key role of graphene as a hot electron active layer that enables the intense EL from silicon-based compound semiconductor LEDs. Such a simple LED structure may find applications in silicon compatible electronics and optoelectronics.

## 1. Introduction

Light-emitting diodes (LEDs) are widely adopted electrically generated light emitting sources and possess vital applications in modern society, specifically III–V based compound semiconductors (GaN, AlGaN, etc.), which are the most commercialized materials for the realization of efficient LEDs [1,2,3,4]. However, the quest for silicon compatible LEDs has huge commercial importance for their applications in monolithic optoelectronic devices and integrated photonic platforms [5,6,7]. To date, effectually competent silicon-based LEDs have not been realized, mainly due to (i) the indirect bandgap of silicon (Si), (ii) issues with the electron mobility of silicon, (iii) comparatively low probability of productive recombination transition in Si quantum cages (Si nanostructures), and (iv) the limited scope of light emission from the seeded Si by foreign light active ingredients [8,9]. In the present work, we have demonstrated the possibility of high intense LEDs by combining Si, double-layer graphene (DLG), and p-type AlGaN [10,11,12].

In recent years, graphene, a two-dimensional (2d) material, has gained huge attention because of its novel superior electronic behavior and has been extensively studied as a highly conductive and transparent electrode for LEDs, and a suitable choice over Indium Tin Oxide (ITO) [13,14,15,16,17]. The double-layer graphene (DLG) is considered to be a better candidate over the monolayer as a substrate and a conductive electrode in device fabrication because DLG possesses better mechanical properties, which can avoid the misconceptions caused by damage or discontinuity in monolayer graphene [18]. Herein, we have presumed DLG as an interfacial layer in between the Si/dielectric layer and p-AlGaN layer to realize a unique illumination mechanism for better performance of LEDs. Recently, the twisted double-layer graphene (DLG) has been demonstrated to be superconducting at low temperatures [19]. Furthermore, as graphene has a zero band gap, the hot electron can experience multi electron-electron interaction before cooling in the time scale of several tens or hundreds of fs, which results in the effect of carriers multiplication (CM) been proven by many reports [20,21]. 

For Si and III–V compound semiconductors, a well-defined epitaxial growth of multiple quantum wells or heterostructure is needed to achieve efficient LEDs, and fabrication of such delicate heterostructures require molecular beam epitaxy (MBE) or metal-organic chemical vapor deposition (MOCVD) exclusively [22,23,24]. The van der Waals integration method [25,26] is a simple and fast method to achieve material integration. It allows for device design flexibility because it involves the direct physical assembly of pre-building blocks of the device. We have followed the van der Waals integration method to realize the silicon-based compound semiconductor heterostructured LEDs: (i) n-Si/HfO_2_(50 nm)/DLG/p-AlGaN LED, (ii) n-Si/SiO_2_(90 nm)/DLG/p-AlGaN LED, (iii) graphene free n-Si/HfO_2_(50 nm)/p-AlGaN LED, (iv) graphene free n-Si/SiO_2_(90 nm)/p-AlGaN LED, and (v) graphene free n-Si/Al_2_O_3_(10 nm)/p-AlGaN LED. We carried out a comprehensive EL study of these LEDs and emphasized the key role of DLG for the illumination mechanism of LED at forward bias voltages. The van der Waals integrated silicon-based LED with graphene as an interfacial layer may have considerable applications in the field of silicon compatible integrated optoelectronics.

## 2. Materials and Methods

The fabrication of the n-Si/HfO_2_/DLG/p-AlGaN LED was done using the van der Waals integration method. The n-Si/HfO_2_ (50 nm) heterostructure was physically attached to the DLG/p-AlGaN heterostructure. This physically attached combination was clamped tightly, resulting in the completion of the fabrication of the LED. The device area is typically 0.5 cm × 0.5 cm. The fabrication of other LEDs was also accomplished similarly as above-mentioned. The p-AlGaN layer with a thickness of 800 nm was grown on a pre-designed AlN/Sapphire substrate via MOCVD (Supplementary Materials). Graphene was prepared on the Cu foils using the chemical vapor deposition (CVD) method and the temperature profile of the CVD growth of polycrystalline graphene is shown in Figure S1. DLG was transferred onto the surface of the p-AlGaN by following the PMMA assisted layer-by-layer graphene transfer method (detailed in the Supplementary Materials). The Au/Cr and Au/Ni electrodes were achieved on the edge of the n-Si and p-AlGaN, respectively, via the electron beam evaporation technique and followed by annealing at 600 °C under an inert atmosphere for better electrical contacts (Supplementary Materials).

Cross-sectional samples of the n-Si/HfO_2_ heterostructure was obtained using a Dual-beam FIB (Quanta 3D FEG, FEI), which were further characterized via high-resolution Transmission Electron Microscope (TEM) images and scanning TEM-x-ray energy dispersive spectra (STEM-EDX) mapping inside a TEM (FEI-TITAN operating at 200 kV). Raman spectra on graphene was collected using a Renishaw Micro Raman Instrument (Figure S2), with a laser source of 532 nm and a focus diameter of 5 µm. Figure S3a shows the X-ray diffraction (XRD) patterns for (atomic layer deposition) the ALD-HfO_2_ thin film (50 nm) on silicon. The XRD spectra were collected on a BRUKER-XRD system by using the Cu Kα line (1.5402 A^o^ of wavelength). Figure S3b shows the Raman spectra of the SiO_2_(90 nm)/Si heterostructure. The current–voltage (I–V) characteristics of the vertical heterostructured graphene-based n-Si/HfO_2_(50 nm)/DLG/p-AlGaN LED, n-Si/SiO_2_(90 nm)/DLG/p-AlGaN LED, and graphene free LEDs were measured by using a Keithley 2400 source meter in forward bias. The electroluminescence (EL) characterization was carried out by using an Ocean Optics QE Pro.

## 3. Results and Discussion

**Figure 1:**
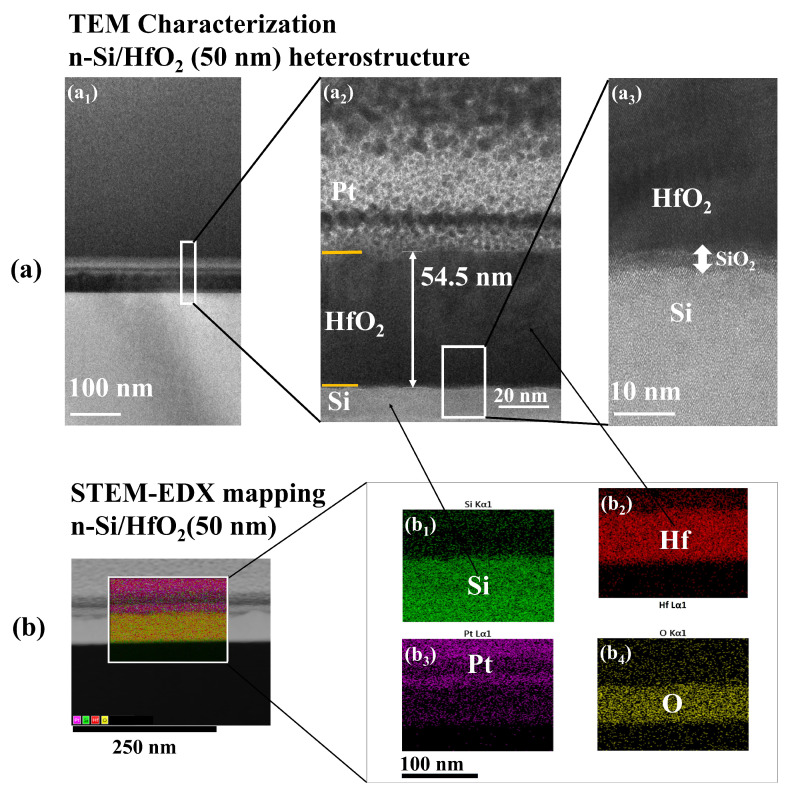
(**a**) Cross-sectional high-resolution Transmission Electron Microscope (HR-TEM) image of the interface of n-Si/HfO_2_ heterostructure. (**a_1_**) Transmission Electron Microscope (TEM) image of cross-sectional view of the interface of n-Si/HfO_2_ with 100 nm scale. (**a_2_**) and (**a_3_**) TEM magnified view of the interface of n-Si/HfO_2_, from which a thickness of 54.5 nm for the HfO_2_ layer, SiO_2_ interface layer, and a hetero-layered stacking of Si and HfO_2_ can be read out respectively. (**b**) STEM-EDX elemental mapping of (**b_1_**) Si, (**b_2_**) Hf, (**b_3_**) Pt, and (**b_4_**) O in the hetero-layered structure of n-Si/HfO_2_.

Figure 1a shows the cross sectional HR-TEM image of the interface of n-Si/HfO_2_ heterostructure. Figure 1a_1_ shows the TEM image of the interface of the vertical heterostructured n-Si/HfO_2_ (cross-sectional view) at the 100 nm scale. Figure 1a_2_,a_3_ show the TEM images of the magnified cross-sectional view of the interface of the n-Si/HfO_2_ heterostructure, from which a thickness of 54.5 nm for the HfO_2_ layer and a hetero-layered stacking of Si and HfO_2_ was read out respectively. The atomic layer deposition (ALD) growth of HfO_2_ on the Si accompanied the formation of SiO_2_ of a few nanometers thick as an interfacial layer between Si and HfO_2_ layers, as shown in Figure 1a_3_ [27]. Figure 1b shows the STEM-EDX elemental mapping of (b_1_) Si, (b_2_) Hf, (b_3_) Pt, and (b_4_) O in the hetero-layered structure of n-Si/HfO_2_.

**Figure 2:**
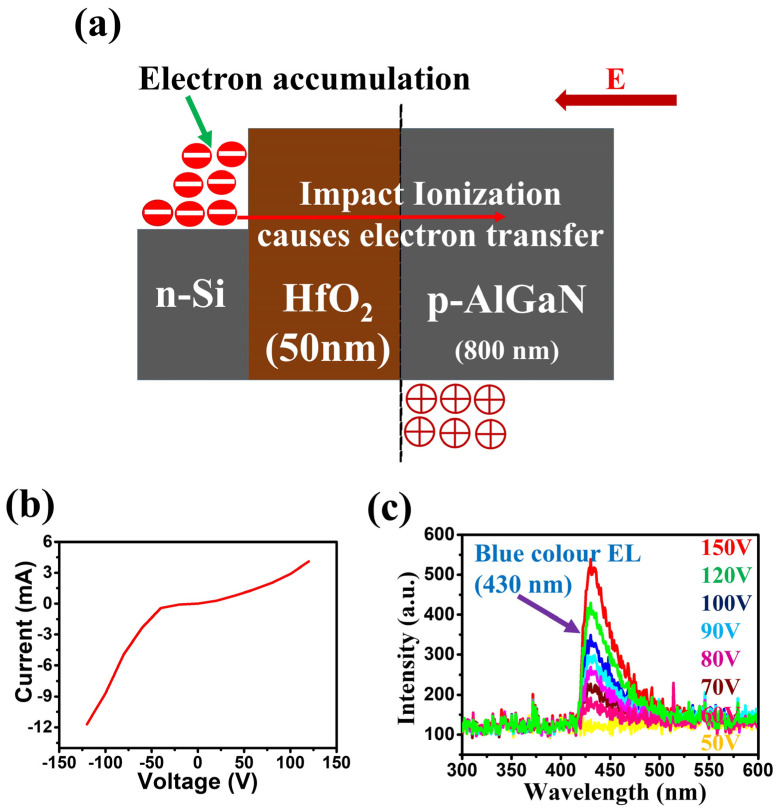
(**a**) Pictorial view of the graphene free n-Si/HfO_2_/p-AlGaN LED. (**b**) Current-Voltage (I–V) characteristic curve of the LED. (**c**) Electroluminescence (EL) of the LED at forward bias applied voltages.

Figure 2a–c shows the schematic view of the graphene free n-Si/HfO_2_(50 nm)/p-AlGaN LED, I–V characteristics of the LED, and EL chart of the LED. As shown in Figure 2a, in the n-Si/HfO_2_(50 nm)/p-AlGaN LED, the hot electrons accumulate at the interface of the n-Si/HfO_2_(50 nm) heterostructure under forward bias [28]. With a relatively larger thickness of the dielectric layer (50 nm, 90 nm), the electron tunneling mechanism will be marginal and the electron-impact ionization mechanism would be the prime contributor for the electron transfer through the dielectric layer.

Electrically induced high energy hot electrons can strongly interact with the lattice of the dielectric layer, causing the emission of more electrons and these electrons can transfer to the p-AlGaN, but the Coulomb scattering of these charges at the p-AlGaN leads to electron–lattice interactions resulted in a predominantly unproductive recombination (heat as major outcome), hence merely a detectable blue color very low intensity EL (wavelength 430 nm) was observed from the LED as shown in Figure 2c. Figure S4a shows the I-V curve of the graphene-free n-Si/Al_2_O_3_(10 nm)/p-AlGaN LED with a 10 nm thickness of the dielectric layer, and at forward bias a low intensity EL with 430 nm (blue light) along with a stumpy peak of defect based EL was observed from the LED, as shown in Figure S4b.

**Figure 3:**
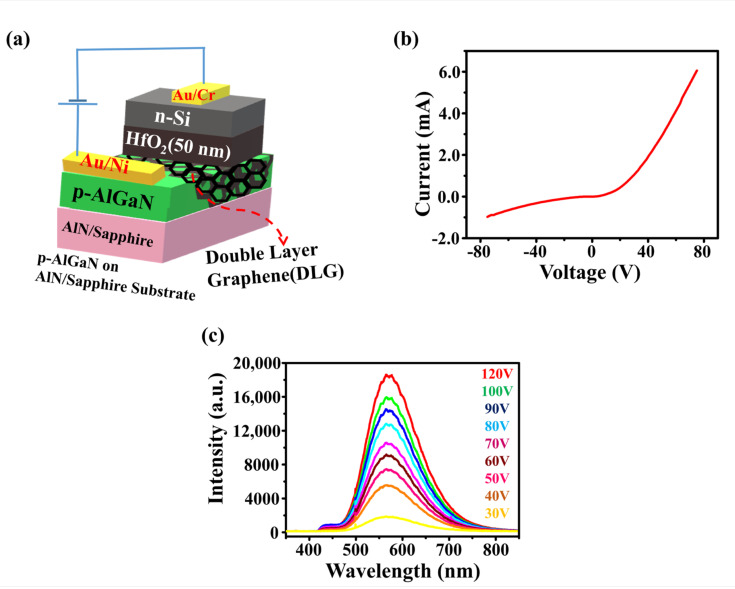
(**a**) Structure of n-Si/HfO_2_/DLG/p-AlGaN LED. (**b**) Current-Voltage (I–V) characteristics of the LED. (**c**) Electroluminescence (EL) of the LED at applied forward bias voltages. Defects based broadband yellow color with peak center at 580 nm.

**Figure 4:**
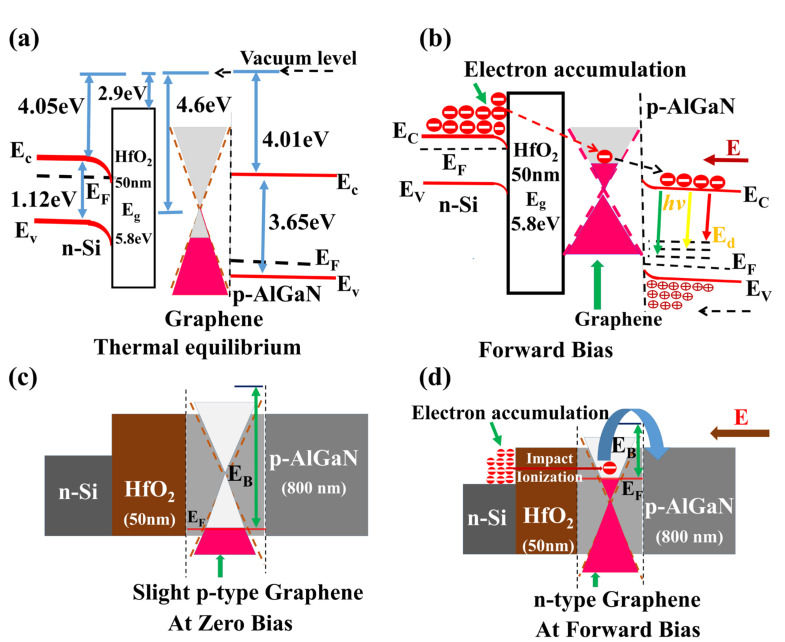
(**a**) Energy band profile of n-Si/HfO_2_/DLG/p-AlGaN vertical semiconductor heterostructure under zero bias condition. (**b**) The illumination mechanism of the LED at forward bias voltages. (**c**) Pictorial view of the electronic band profile of graphene in the LED at zero bias. (**d**) Pictorial view of the band profile of graphene in the LED at forward bias. Electric field induced hot electron accumulation at the graphene led to the n-type doped graphene and rise in Fermi level of the graphene.

Figure 3a–c shows the device structure of n-Si/HfO_2_(50 nm)/DLG/p-AlGaN LED, I–V characteristics of the LED, and EL of the LED in forward bias, respectively. Figure 4a,b shows the energy band profile of the n-Si/HfO_2_(50 nm)/DLG/p-AlGaN LED under zero bias and at forward bias, respectively. In the n-Si/HfO_2_(50 nm)/DLG/p-AlGaN LED, at zero bias, DLG displayed a slight p-type nature, as shown in Figure 4c. The unavoidable presence of metal ions, the residues of Polymethyl methacrylate (PMMA) on the graphene, and the transfer of graphene onto the AlGaN all together led to the slight p-type doped DLG [29,30]. The transfer of DLG onto the p-AlGaN resulted in the formation of an energy barrier (E_B_) for the electron transfer from graphene to the p-AlGaN at the interface of DLG/p-AlGaN and the slight p-type behavior of DLG and intrinsic polarization effects of p-AlGaN caused the Schottky effects, and ascended the energy barrier E_B_ between DLG and the p-AlGaN, as shown in Figure 4c [31,32,33,34].

At applied forward voltages, the electric field induced hot electrons accumulated at the interface of the n-Si/HfO_2_ heterostructure can cross the thick dielectric barrier (50 nm) by the electron-impact ionization mechanism [35,36,37,38] and are injected into the graphene. The superior electronic behavior of graphene [39,40,41,42,43] (like strong electron–electron interactions, high electrical conductivity along the plane, high charge carrier mobility, and weak charge carrier scattering, etc.) enables the accumulation of hot electrons at the graphene, which may amplify the numbers of electrons through multiplication exciton effect.

The accumulation of electrically induced hot electrons at the graphene [44,45] (resistant to the extrinsic scattering of electrons) led to the n-type doped graphene or a rise in the Fermi level of graphene, which reduces the energy barrier (E_B_) between the graphene and p-AlGaN, resulting in the significant electron transport from graphene to the p-AlGaN, as shown in Figure 4d [46]. Hence, the excess of negative charge or numerous hot electrons available at the graphene can be effectively transferred into the p-AlGaN, as shown in Figure 4b,d [47,48,49], which leads to the radiative recombination of holes with the readily available hot electrons at the p-AlGaN, which resulted in a defect based strong EL or high-intensity yellow color with the center of the peak at 580 nm, as shown in Figure 3c, where a further increase in the forward applied voltage increased the intensity of the yellow color illumination. Experimentally no significant illumination or very low intensity EL was observed without the presence of graphene from the LED, as shown in Figure 2c and Figure S4. The accumulation of hot electrons at the graphene is the key factor for the illumination of intense EL from the LED.

**Figure 5:**
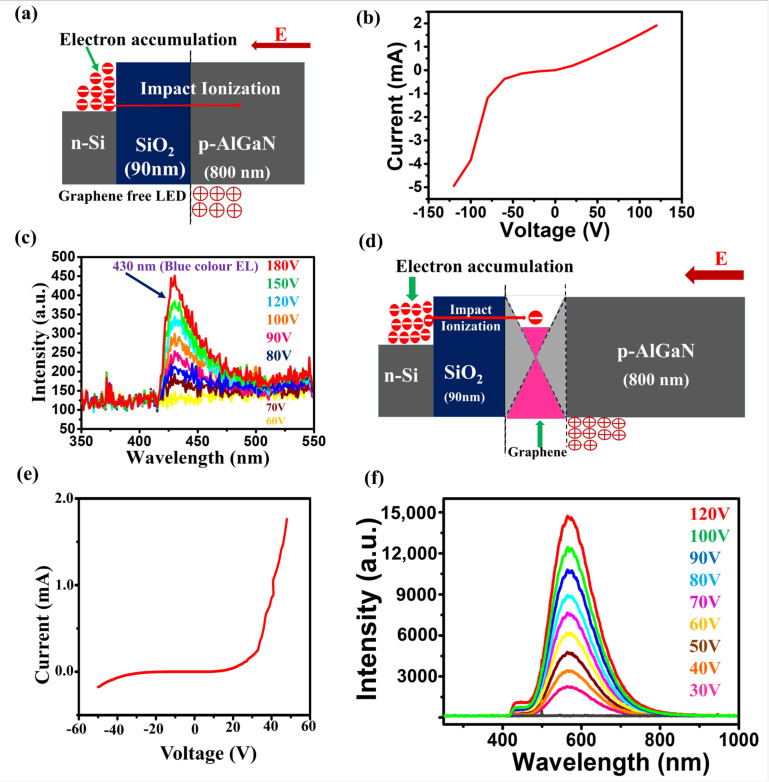
(**a**) Schematic representation of the structure of graphene free n-Si/SiO_2_/p-AlGaN LED. (**b**) Current-Voltage (I–V) curve of the graphene free LED. (**c**) Electroluminescence (EL) of the graphene free LED at forward bias applied voltages. (**d**) Pictorial view of the graphene based Si/SiO_2_/DLG/AlGaN LED. (**e**) I–V curve of the graphene based LED. (**f**) EL from the graphene based LED in forward bias. The illumination from the graphene based LED was a defect based broadband yellow color with a wavelength maxima (λ_max_) at 580 nm.

Figure 5a–c shows the graphene free n-Si/SiO2(90 nm)/p-AlGaN LED, I–V curve of the LED, and the EL from the LED at forward bias applied voltages, respectively. There was no detectable light emission from the graphene free n-Si/SiO2(90 nm)/p-AlGaN device at moderate applied voltages (below 70 V), but at very high applied voltages (about 100 V), a blue colored very weak EL (430 nm) was observed (Figure 5c). At forward applied voltages, the illumination mechanism in the graphene free silica dielectric based LED was similar to the graphene free n-Si/HfO2(50 nm)/p-AlGaN LED. The electrically induced hot electrons accumulated at the interface of n-Si/SiO2 can cross the thick dielectric barrier via electron-impact ionization, but due to the scattering of charge carriers and electron–lattice interactions at the p-AlGaN, unproductive recombination would predominate, which leads to the very low intensity EL (blue color).

Figure 5d and Figure S5 both show the schematic illustration of the n-Si/SiO_2_(90 nm)/DLG/p-AlGaN LED, while Figure S6a,b shows the electronic band alignment at zero bias and the illumination mechanism of LED at forward bias, respectively. Figure 5e shows the I–V characteristics of the LED. We made an enthusiastic study regarding the influence of the thickness of the dielectric on the EL from the LED. The light emission mechanism of the n-Si/SiO_2_(90nm)/DLG/p-AlGaN LED was similar to that of the n-Si/HfO_2_(50nm)/DLG/p-AlGaN LED. In the n-Si/SiO_2_(90 nm)/DLG/p-AlGaN LED, under forward voltages, the electron-impact ionization mechanism enables the transfer of hot electrons through the thick dielectric layer, and the electrically induced hot electron accumulation at the graphene caused the rise in the Fermi level of graphene and reduction in the E_B_, which led to the efficient electron transport from graphene to the p-AlGaN. The readily available hot electrons at the p-AlGaN recombined with the holes, resulting in a defect based broadband high-intensity yellow color emission, as shown in Figure 5f. In a comparative EL study between HfO_2_ (50 nm) and SiO_2_ (90 nm) dielectric layer based graphene LEDs, the moderately lower EL from the silica dielectric based graphene LED, with no drastic EL variations infers the vital role of the electron-impact ionization mechanism. The comparative EL study between the graphene free and graphene based LEDs at the different thickness of the dielectric layer revealed that even though the impact ionization was a necessary step for electron transfer through the dielectric layer, it was not a crucial influential factor for the intense EL from the LED at the respective thickness of the dielectric layer, and it highlights the graphene as a key component for the illumination mechanism of the LED.

Graphene (G), as an interfacial layer between two semiconductors (S), acts as an electric field induced hot electron accumulation layer, and displays an n-type nature, favoring the efficient electron transfer from graphene to the semiconductor, which may have significant applications in S-G-S (S-M-S) type hot electron based electronic and optoelectronic devices. Van der Waals integration makes possible a wide variety of S-G-S combinations.

## 4. Conclusions

In conclusion, we fabricated the n-Si/HfO_2_(50 nm)/DLG/p-AlGaN LED and n-Si/SiO_2_(90 nm)/DLG/p-AlGaN LED by using the van der Waals integration method. In forward applied bias voltages, the silicon/dielectric layer (HfO_2_, SiO_2_) heterostructure accumulates numerous hot electrons at the interface of the Si/dielectric layer, and these electrically induced hot electrons are capable of crossing the thick dielectric barrier via electron-impact ionization mechanism and can be injected into the graphene. The superior electronic behavior of graphene alike strong electron–electron interactions, high conductivity along the plane, etc., leads to the accumulation of hot electrons at the graphene, and CM effect in graphene multiply the numbers of electrons and eventually transfers to p-AlGaN and contribute to the emission. The accumulation of hot electrons at the graphene results in a rise in the Fermi level of graphene, which also reduces the energy barrier E_B_ for electron transfer from graphene to the AlGaN. Hence, at applied forward voltages, the excess hot electrons injected into the graphene can effectively cause electron multiplication and efficiently transfer into p-AlGaN, herein holes recombine with these hot electrons at a significant rate, causing the broadband yellow color EL with a center peak at 580 nm.

Experimentally, the graphene free n-Si/HfO_2_(50 nm)/p-AlGaN LED and n-Si/SiO_2_(90 nm)/p-AlGaN LED showed a blue color, merely detectable, very low intensity EL (430 nm), even at very high applied forward voltages (120 V). The graphene based n-Si/dielectric layer (HfO_2_-50 nm, SiO_2_-90 nm)/DLG/p-AlGaN LED structures both showed high EL at applied forward voltages. Comparatively, no extreme change in the intensities of EL from the graphene based LEDs was observed, which specifies the significant role of the electron-impact ionization mechanism for electron transfer through the thick dielectric layer. The DLG is the key component, acts as a hot electron accumulator, and plays a vital role in the illumination of the LED. The Si/dielectric layer (HfO_2_, SiO_2_ with optimized thickness) heterostructure is necessary for a strong, high EL of the LED. The present Si/dielectric layer/graphene/AlGaN based LED demonstrates a unique device design and the importance of graphene for a better performance of silicon-based LEDs, which may find significant applications in the field of silicon compatible optoelectronics.

## Supplementary Materials

The following are available online at https://www.mdpi.com/2079-4991/10/12/2568/s1, Figure S1: CVD growth of graphene, Figure S2: The Raman spectra of graphene, Figure S3: XRD pattern of the 50nm HfO_2_ on n-type Si, and Raman spectra of n-type Si/SiO_2_ heterostructure respectively, Figure S4: Graphene free Si/Al_2_O_3_(10 nm)/AlGaN LED, Figure S5: Pictorial representation of the Si/SiO_2_(90 nm)/DLG/AlGaN LED, Figure S6: Illumination mechanism of the Si/dielectric layer(SiO_2_)/DLG/AlGaN LED.
