# Supplementary material for: Van der Waals Integrated Silicon/Graphene/AlGaN Based Vertical Heterostructured Hot Electron Light Emitting Diodes
Source: Nanomaterials (Basel). 2020 Dec 21;10(12):2568. doi: 10.3390/nano10122568 (PMC7767542; doi:10.3390/nano10122568)
Supplement: Supplementary file 1 [file nanomaterials-10-02568-s001.pdf]

Supplementary Materials

Van der Waals Integrated Silicon/Graphene/AlGaN Based Vertical Heterostructured Hot Electron Light Emitting Diodes

Nallappagari Krishnamurthy Manjunath [1], Chang Liu[1], Yanghua Lu [1], Xutao Yu [1] and Shisheng Lin[1,2,*]

1 College of Microelectronics, College of Information Science and Electronic Engineering, Zhejiang University, Hangzhou, 310027, China; manjunath@zju.edu.cn

2 State Key Laboratory of Modern Optical Instrumentation, Zhejiang University, Hangzhou, 310027, China; shishenglin@zju.edu.cn

* Correspondence: shishenglin@zju.edu.cn; Tel.: +86-0571-87951555

Supplementary Note

1. Fabrication of AlGaN layer on Sapphire

An AlGaN layer with an Mg concentration of 5 x10^{19} cm^{-3} and a thickness of 800 nm was grown on the sapphire substrate by metal-organic chemical vapor deposition (MOCVD). The hole concentration is about 5×10^{17} cm^{-3} for the AlGaN layer (through Hall effect measurement of the hole concentration). After ultrasonic cleaning in acetone, ethanol, and DI water respectively for 5 min, the samples were dried by N_2. Subsequently, Ni (20 nm)/Au (50 nm) contacts were achieved by the electron-beam evaporation method onto one end of the sample and followed by intermediate annealing at 600°C in N_2 conditions for 30 min to reduce the contact resistance. [1]

2. CVD growth of graphene

Continuous films of polycrystalline Graphene (monolayer and bilayer) were primarily grown on a few micrometer thick Cu foils (Alfa Aesar). The polished Cu or moisture-free Cu samples were cut into 5 cm length and 2cm width strips and positioned in a hot wall furnace consisting of a 22-mm ID fused silica tube heated with a split tube furnace. A typical growth process of graphene flow is (1) load the fused silica tube with the Cu foil, evacuate, backfill with hydrogen, heat to 1000°C, and maintain H_2(g) pressure of 69 Pa under a 40 sccm flow; (2) stabilize the Cu film at the desired temperatures, up to 1000℃, and introduce 5 sccm of CH_4(g) for the desired period of 45 minutes at a total pressure of 92 Pa; (3) after exposure to CH_4, the furnace was cooled to room temperature. The experimental parameters (temperature profile, gas composition/flow rates, and system pressure) are shown in Fig. S1. The cooling rate was varied from 300℃/min to about 400℃/min which resulted in films with no discernable differences.

Graphene films were removed from the Cu foils by etching in an aqueous solution of iron nitrate or the ferric chloride. The etching time was found to be a function of the etchant concentration, the area, and thickness of the Cu foils. The PMMA method was used to transfer the graphene from the Cu foils. The surface of the graphene-on-Cu is coated with poly-methyl methacrylate (PMMA) and after the Cu is dissolved, and the PMMA/graphene is lifted from the solution. [2] The graphene films are easily transferred to other desired substrates such as the TEM grid, SiO_2/Si, sapphire, SiC, etc. with significantly fewer holes or cracks (< 5% of the film area).

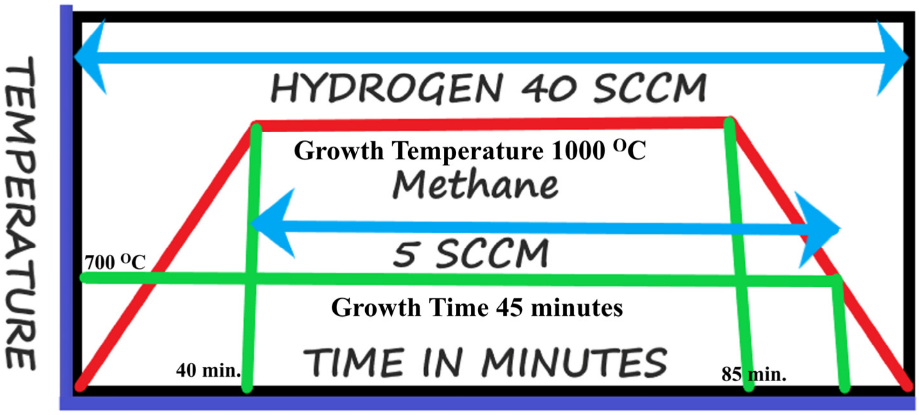

Figure S1. CVD growth of graphene. The temperature profile of the CVD growth of polycrystalline graphene. (continuous films of monolayer and bilayer graphene).

3. Fabrication of Graphene/AlGaN heterostructure.

Double layered graphene was prepared by layer-by-layer transfer method. Graphene/Cu was used as a substrate to lift the graphene/polymethylmethacrylate (PMMA) membrane floating on the DI water. After that the sample allowed for naturally drying, then it was kept for annealing at about

105°C for 30 min. This sample was further treated for standard PMMA assisted graphene transfer method to obtain graphene/graphene/PMMA (bilayer graphene/PMMA) membrane. Then, the PMMA supported double-layer graphene was transferred onto the p-AlGaN and washed with acetone, and kept for drying. [3,4] The thickness of the double layer was expected to be 0.81 nm. [5]

4. Characterization, electrical and optical measurement.

Raman spectra were measured by a Renishaw micro Raman spectrometer with an excitation laser of 532 nm. The current-voltage (I-V) characteristics were measured by a Keithley 2400 multimeter. Electroluminescence (EL) spectra were recorded by a fiber optic spectrometer (Ocean view, QE pro). The Raman spectrum of graphene is shown in Fig. S2. The thickness of the n-Si was about 400 μm. The XRD spectrum of Si/HfO_2 and Raman spectrum of Si/SiO_2 are shown in Fig. S3. The Fig. S4a and S4b shows the I-V curve of the graphene free Si/Al_2O_3(10 nm)/AlGaN LED, and the EL of the LED at forward bias applied voltages respectively. The schematic illustration of the device structure of Si/SiO_2/DLG/AlGaN LED is shown in Fig. S5. The energy band diagram and illumination mechanism of Si/SiO_2/DLG/AlGaN LED are shown in Fig. S6.

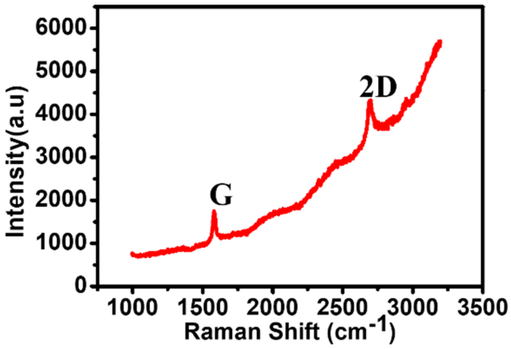

Figure S2. The Raman spectra of graphene. In the Raman characterization, graphene as excited with a laser of 532 nm, the Stokes phonon energy shift triggered by laser excitation generates two main peaks in the Raman spectrum. Graphene can be recognized by the location and character of its G (1580 cm^{-1}) and 2D (2690 cm $^{-1}$) peaks.

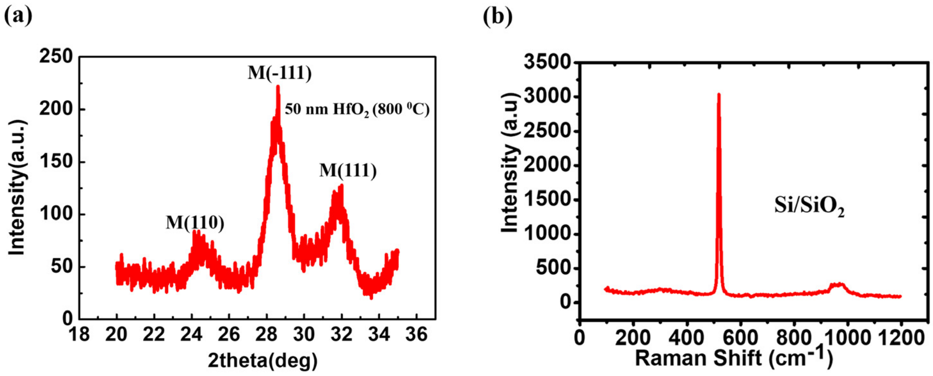

Figure S3. Silicon/dielectric layer hetero-structures. (**a**) XRD pattern of the 50nm HfO_2 on n-type Si. (**b**) Raman spectra of n-type Si/SiO_2 heterostructure.

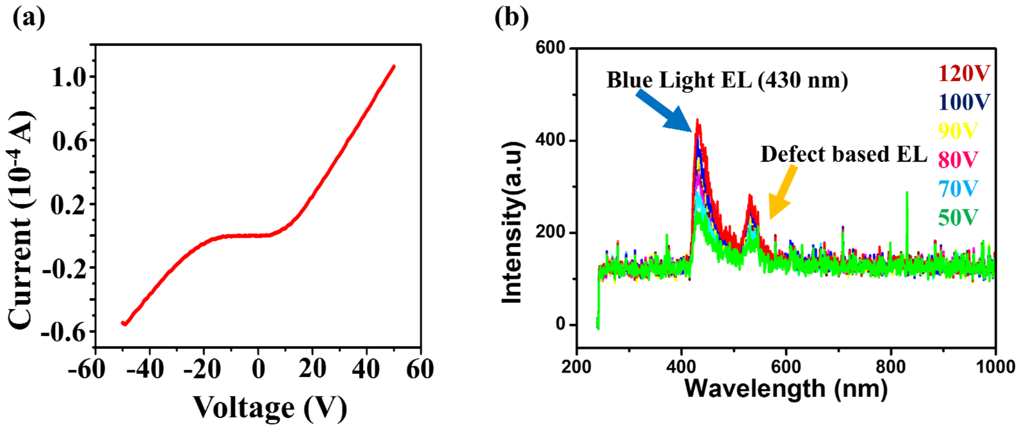

Figure S4. Graphene free Si/Al₂O₃(10 nm)/AlGaN LED. (**a**) I-V curve of the n-Si/Al_2O_3(10 nm)/p-AlGaN LED (**b**) EL of the LED in forward bias (EL at 430 nm along with defects based very weak illumination).

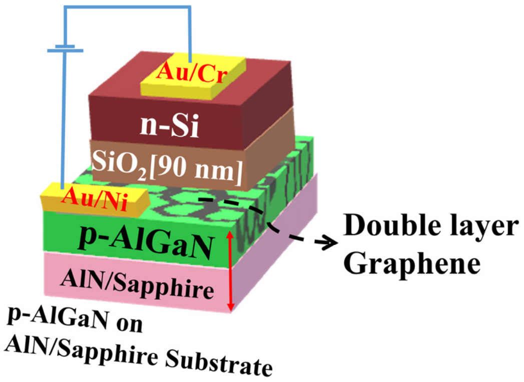

Figure S5. Silicon/graphene/AlGaN LED. Pictorial representation of fabricated n-Si/SiO_2/DLG/p-AlGaN LED.

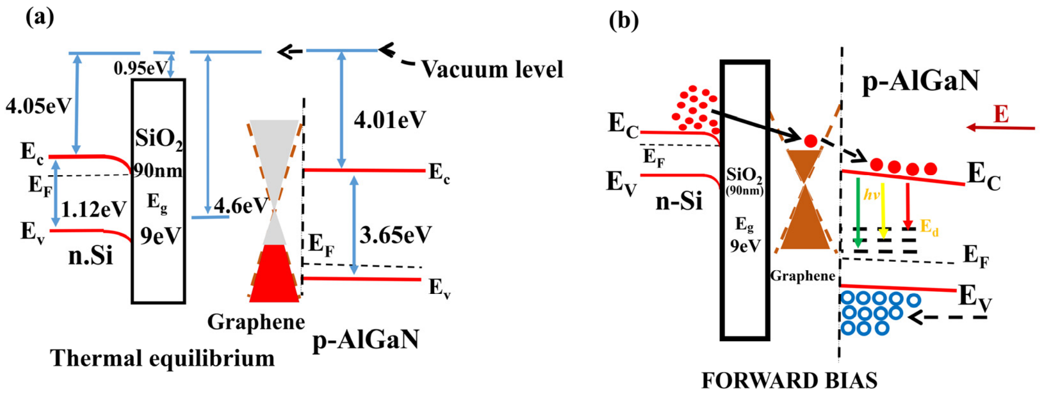

Figure S6. Illumination mechanism of silicon/dielectric layer(SiO_2)/DLG/AlGaN LED. (**a**) Energy band profile of the n-Si/SiO_2/DLG/p-AlGaN vertical semiconductor hetero-structure under (zero bias)

thermal equilibrium conditions. (**b**) The illumination mechanism of the n-Si/SiO_2/DLG/p-AlGaN LED at forward bias voltages. Defects based broadband yellow color illumination from LED with wavelength maxima (λ_{max}) at 580 nm.

References

[1] Liu Z. H, Ng G. I, Zhou H, Arulkumaran S, and Maung Y. K. T. Reduced surface leakage current and trapping effects in AlGaN/GaN high electron mobility transistors on silicon with SiN/Al_2O_3 passivation. Appl. Phys. Lett. 2011; 98: 113506.

[2] Kuo H. K, Sheu J. K, Chi G. C, Huang Y. L, Yeh T. W. Low-resistance Ni/Au ohmic contact to Mg-doped of $Al_{0.15}Ga_{0.85}N$/GaN superlattices. Solid Stat Electron 2001; 45: 717-720.

[3] Li X, Cai W, An J, Kim S, Nah J, Yang D, Piner R, Velamakanni A, Jung I, Tutuc E, Banerjee S. K, Colombo L, Ruoff R. S. Large-area synthesis of high-quality and uniform graphene films on copper foils. Science 2009; 324: 1312-1314.

[4] Dean C. R, Young A. F, Meric I, Lee C, Wang L, Sorgenfrei S, Watanabe K, Taniguchi T, Kim P, Shepard K. L and Hone J. Boron nitride substrates for high-quality graphene electronics. Nature Nanotechnology 2010; 5: 722–726.

[5] Ye S, Shi X, Qi C, and Wang G et al. Thickness-Dependent Strain Effect on the Deformation of the Graphene-Encapsulated Au Nanoparticles. Journal of Nanomaterials 2014; 4: 1-6.
